# Supplementary figures and images for: The spermidine acetyltransferase SpeG regulates transcription of the small RNA rprA
Source: PLoS One. 2018 Dec 18;13(12):e0207563. doi: 10.1371/journal.pone.0207563 (PMC6298664; doi:10.1371/journal.pone.0207563)

**A**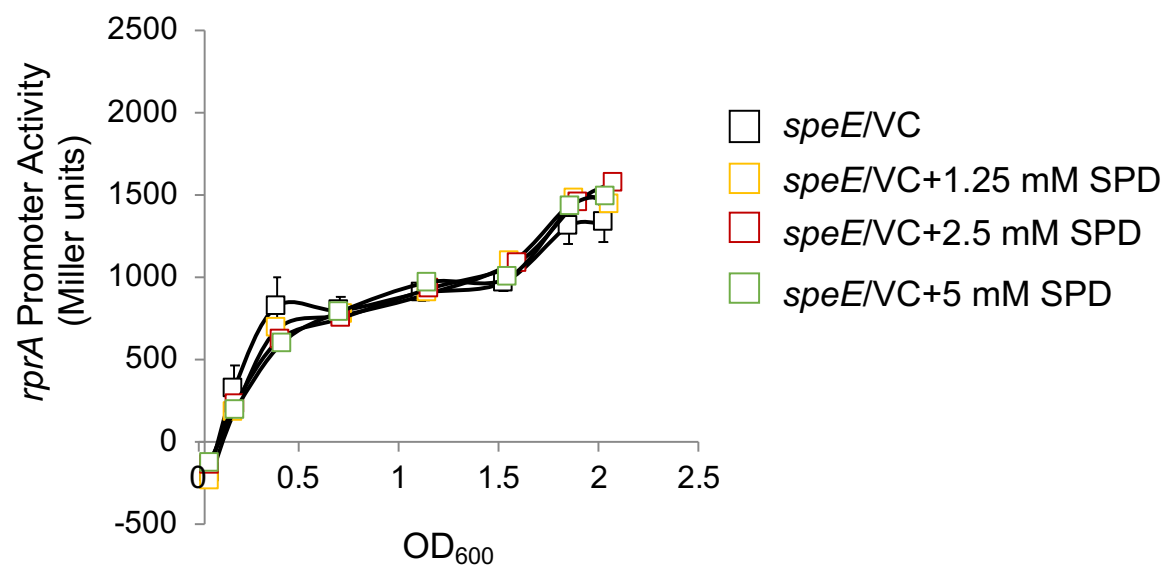**B**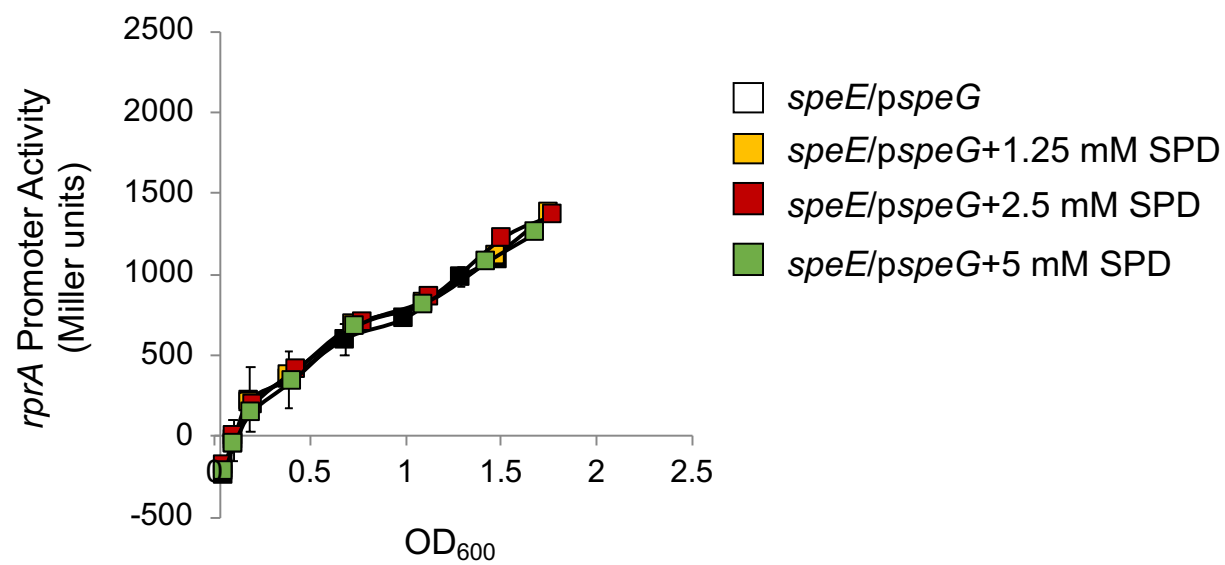

Supplement: S1 Fig — The speE mutant was transformed with either the VC or pSpeG and grown in TB7 supplemented with 50 μM IPTG and 0, 1.5, 2.5, or 5 mM spermidine. Growth and rprA promoter activity was measured over time. Each data point is an average of duplicate biological replicates and standard deviations. (PDF) [file pone.0207563.s001.pdf]

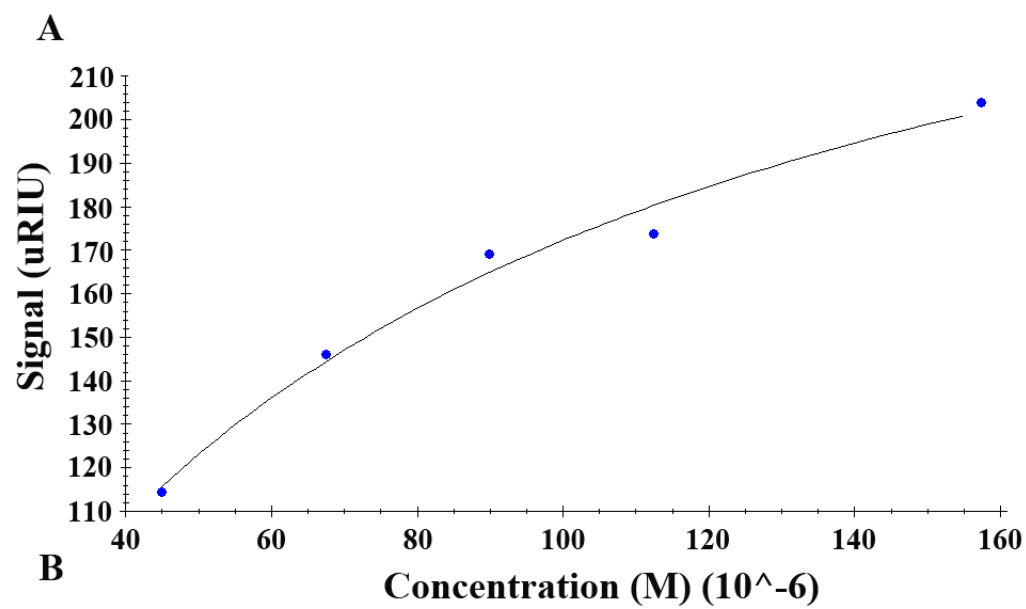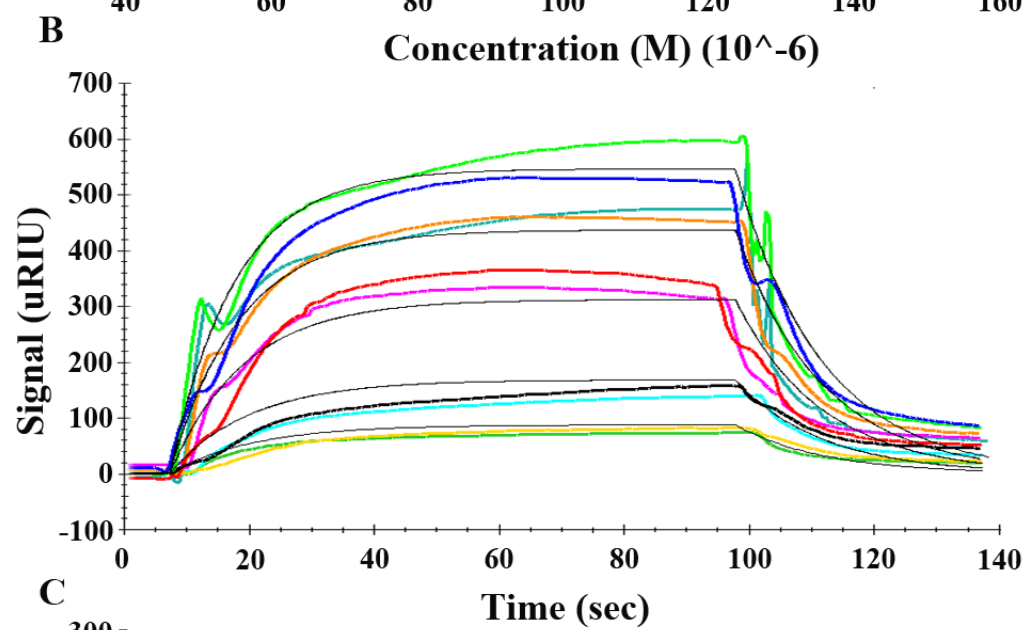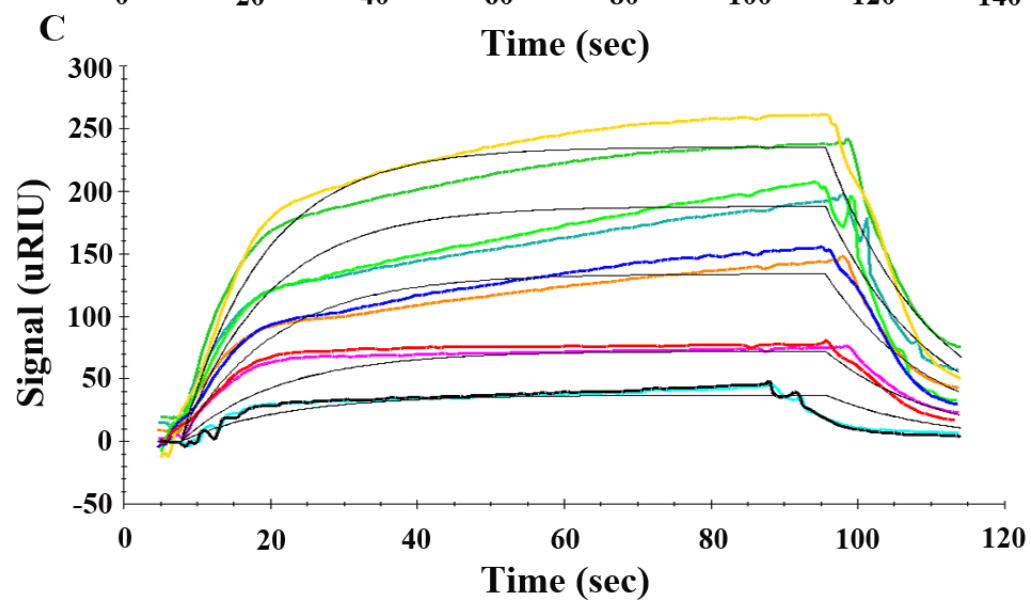

Supplement: S2 Fig — (A) The maximum responses in the SPR sensograms for the first dilution series of RcsB C-terminal domain in the absence of spermidine are plotted against the analyte concentration. (B and C) The SPR sensograms for dilution series of RcsB full-length and its C-terminal domain after exposure to spermidine. The RcsB full-length or RcsB C-terminal domain protein was injected in five dilution series with the following concentrations: 21, 42, 53, 63 and 74 μM (B) or 23, 45, 91, 114, and 136 μM (C). The fitted data are shown in black. (PDF) [file pone.0207563.s002.pdf]

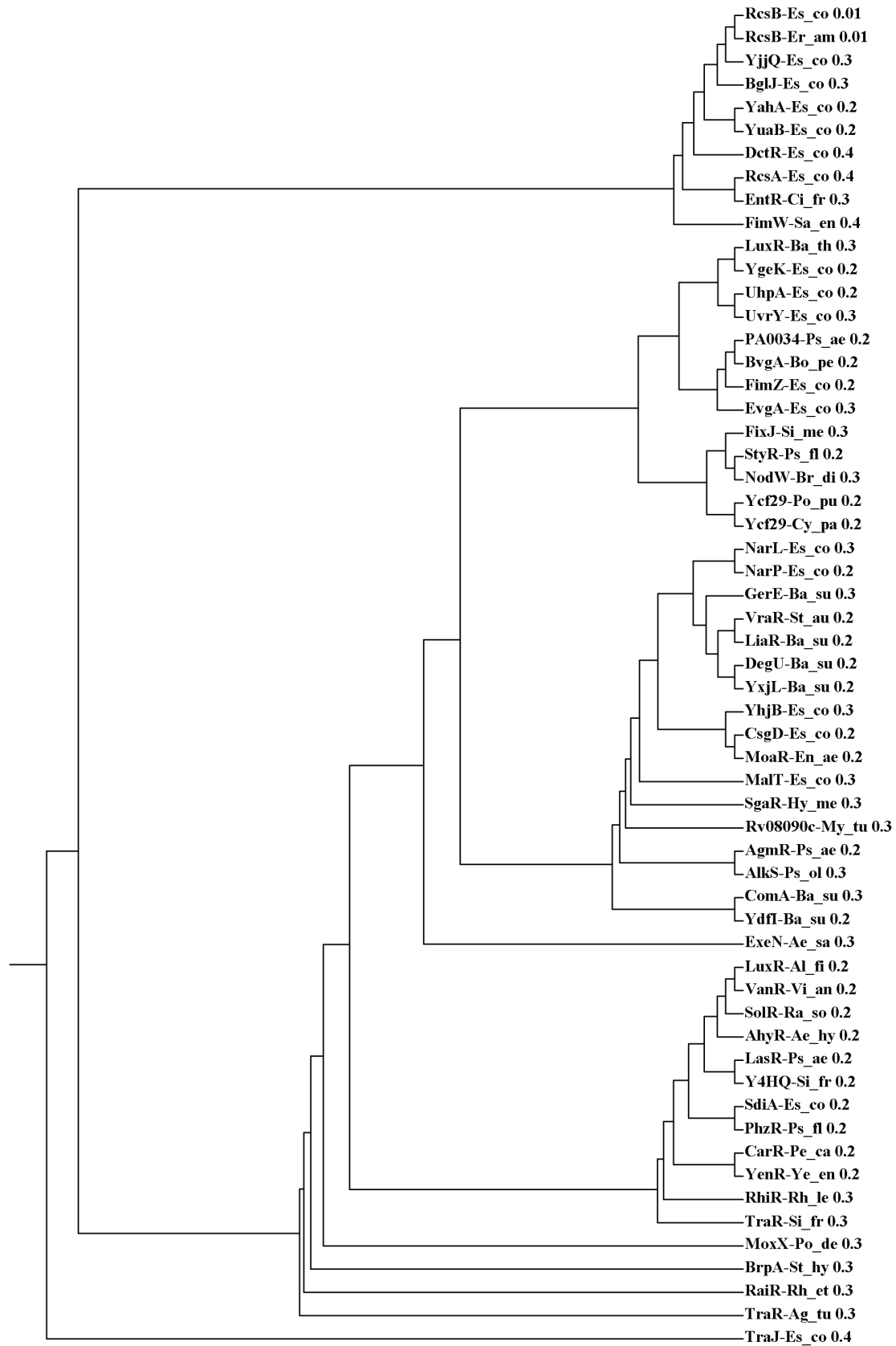

Supplement: S3 Fig — Phylogenetic tree was created in ClustalW2 server (http://www.ebi.ac.uk/Tools/msa/clustalw2). A list of 58 representatives of the conserved LuxR/FixJ DNA-binding domains was generated in NCBI server http://www.ncbi.nlm.nih.gov/Structure/cdd) and includes DNA-binding domains of following transcriptional factors: RcsB from Escherichia coli (RcsB-Es_co) [GI:353570681], RcsB from Erwinia amylovora (RcsB-Er_am) [GI:33357861], YjjQ from E. coli (YjjQ-Es_co) [GI:83288197], BglJ from E. coli (BglJ-Es_co) [GI:3915634], YahA from E. coli (YahA-Es_co) [GI:2506596], YuaB from E. coli (YuaB-Es_co) [GI:81783897], DctR from E. coli (DctR-Es_co) [GI:57012697], RcsA from E. coli (RcsA-Es_co) [GI:60393000], EntR from Citrobacter freundii (EntR-Ci_fr) [GI:6015049], FimW from Salmonella enterica (FimW-Sa_en) [GI:585140], LuxR from Bacteroides thetaiotoamicron (LuxR-Ba_th) [GI:171849138], YgeK from E. coli (YgeK-Es_co) [GI:20140955], UhpA from E. coli (UhpA-Es_co) [GI:84029412], UvrY from E. coli (UvrY-Es_co) [GI:83288180], PA0034 from Pseudomonas aeruginosa (PA0034-Ps_ae) [GI:13959718], BvgA from Bordetella pertussis (BvgA-Bo_pe) [GI:61219948], FimZ from E. coli (FimZ-Es_co) [GI:84028128], EvgA from E. coli (EvgA-Es_co) [GI:82581667], FixJ from Sinorhizobium meliloti (FixJ-Si_me) [GI:159163516], StyR from P. fluorescens (StyR-Ps_fl) [GI:78100993], NodW from Bradyrhizobium diazoefficiens (NodW-Br_di) [GI:128495], Ycf29 from Porphyra purpurea (Ycf29-Po_pu) [GI:1723332], Ycf29 from Cyanophora paradoxa (Ycf29-Cy_pa) [GI:1351750], NarL from E. coli (NarL-Es_co) [GI:24158735], NarP from E. coli (NarP-Es_co) [GI:400374], GerE from Bacillus subtilis (GerE-Ba_su) [GI:13786948], VraR from Staphylococcus aureus (VraR-St_au) [GI:166007196], LiaR from B. subtilis (LiaR-Ba_su) [GI:68051995], DegU from B. subtilis (DegU-Ba_su) [GI:118438], YxjL from B. subtilis (YxjL-Ba_su) [GI:20141933], YhjB from E. coli (YhjB-Es_co) [GI:586682], CsgD from E. coli (CsgD-Es_co) [GI:1706166], MoaR from Enterobacter a [file pone.0207563.s003.pdf]

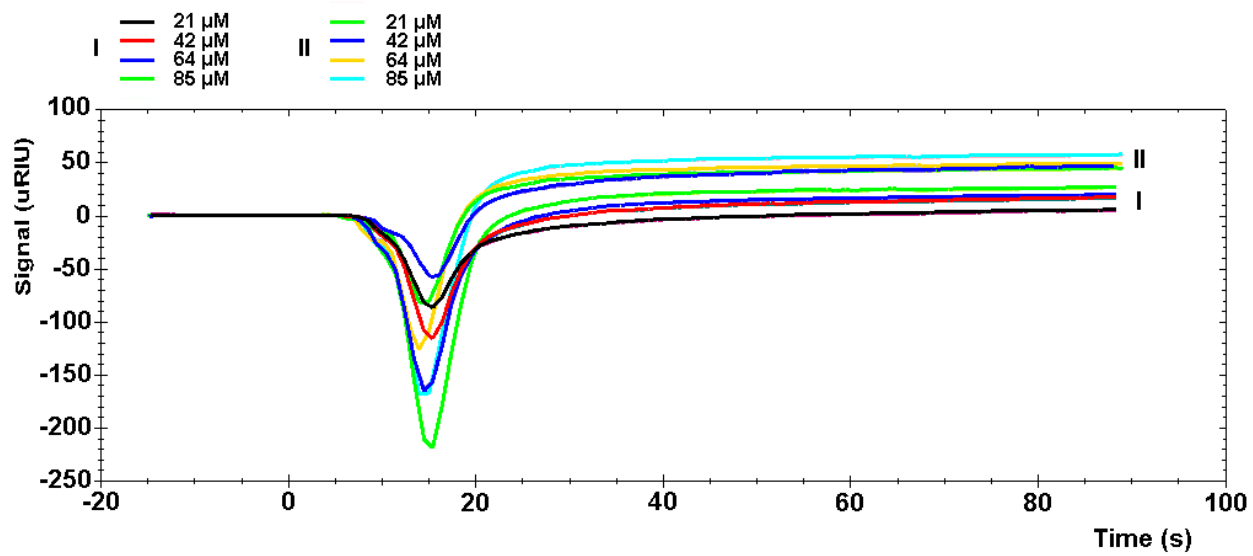

Supplement: S4 Fig — The SPR sensograms of SpeG and transcription regulator RcsA. The RcsA protein was injected in four dilution series. Duplicate measurements for each concentration indicated above SPR sensograms were performed. (PDF) [file pone.0207563.s004.pdf]
